# Fevers

**Published:** 1905-04-15

**Authors:** 


### FEVERS.

(*Continued from page* 32.)

**The Leishman-Donovan Body.**—Evidence is accumulating to show that this organism, the nature of which is not yet determined, is the cause of the identical or allied diseases variously known as cachexial fever, chronic malarial cachexia, kala-azar, human piroplasmosis, tropical splenomegaly, and Dum-dum fever, and also of Oriental sore or Delhi boil. The body in question was first noted by Leishman [8] after death in the spleen of a soldier invalided home to Netley from Dum-dum in 1900 for tropical cachexia and splenomegaly. Subsequently it was found by Donovan both post mortem and in blood withdrawn by splenic puncture during life in similar cases of disease. Many cases have since been recorded by Marchand and Ledingham, Manson and Low, Bentley, Rogers, Christophers, and others. The cases are widely distributed, having been found in many districts of India, in China, Tunis, Algiers, Arabia, Egypt, Assam, and other places. Occasionally Europeans are affected. The symptoms of the disease are:—Marked splenic and usually some hepatic enlargement; long-continued irregularly remittent fever; an earthy pallor or discolouration of the skin; emaciation, often extreme; petechial and other hæmorrhages; transitory œdema of the limbs; complications such as bronchitis, diarrhœa and dysentery, or cancrum oris. Dysenteric diarrhœa is frequent and a common cause of death. The blood examination usually shows a moderate decrease of red corpuscles and hæmoglobin, and a decided decrease of white corpuscles. Rogers [8] states that the normal ratio of 1 white to 625 red corpuscles may fall to 1 to 2,000, or 1 to 4,000, or even lower. It is the polynuclear cells which are especially reduced, they may fall to one-fiftieth, or even one-hundreth, of the normal number, while the lymphocytes, and especially the large mononuclear leucocytes, show a marked relative but not an actual increase. Important negative signs of the disease are the absence of malarial parasites and the failure of quinine medication. The parasite has been likened by Christophers [9] to a closed cockle-shell in general shape. A characteristic feature is the double nucleus, a large oval mass at one periphery of the body and a smaller rod-shaped centrosome on the opposite side. The organism appears to be intracellular. The bluish "zooglea mass," seen surrounding it in stained specimens, is thought by Christophers to be derived from the "macrophages," or large mononuclear cells which contain the parasite and which have been artificially fractured in preparing the blood specimen. Donovan[10] has illustrated an article on this disease, headed "Human Piroplasmosis," by a coloured plate in which the large nucleus and the centrosome and the zooglea masses, together with changes suggesting division and multiplication of the parasite are clearly seen. The organism has not yet been satisfactorily classed. At least three views as to its nature have been advanced. (1) Ross, working on films sent him by Donovan, concluded that the bodies represented a new genus belonging to the sporozoa, which he named *Leishmania.* It is interesting to note in connection with this view that the bodies found by Wright in Delhi boil,[11] which, if not identical with, are nearly related to the parasites found in Kala-azar and chronic malarial cachexia, were by him regarded as a new genus, and named "helcosama." (2) Laveran, who also examined films sent by Donovan, decided that the parasites were contained within the red corpuscles, and therefore belonged to the piroplasmata. He suggested the name *P. Donovani.* Other observers have not found the parasites within the red corpuscles, and do not corroborate this theory. (3) Leishman's own view, in which he is supported by Marchand, Ledingham, and Rogers, is that the organisms represent a stage in the life history of a flagellate, either a trypanosoma, or a member of a closely allied genus. This view has been questioned, but observers such as Schaudinn[12] and Prowazek have described, in tracing the life history of true trypanosomes, the development of forms very similar to the Leishman-Donovan body. And quite recently[13] Rogers has reported the development of fully developed trypanosomata in a case of cachexial fever, and also in one of Kala-azar on incubating at 22° C. the citrated blood of the patients withdrawn by splenic puncture. If these observations of Rogers are confirmed Leishman's contention will be established. The mode of entrance and of exit of the body are unknown at present, but the frequent association of intestinal symptoms, and the fact that the bodies have been repeatedly found in the intestinal ulcers, suggest their elimination by the bowel. The researches of Schaudinn[14] above mentioned into the developmental changes undergone by trypanosomes, make it extremely likely that the spirillum of relapsing fever, the *Spirochæte Obermeieri,* which has hitherto been regarded as a bacterium, is in reality a protozoan parasite, and a phase of a trypanosome.

**Trypanosome Diseases.**—The subject of trypanosomiasis is daily becoming one of more absorbing interest not only from the purely medical standpoint but also on account of the vast economic problems involved in its occurrence. The latter aspects of the question have recently been touched on by Sir Charles Bruce in his Introductory Address to the London School of Tropical Medicine.[15] During his career as governor of several of our colonies Sir Charles has had to face many epidemics of malaria, plague, and other tropical diseases, while in 1902 Mauritius was threatened with commercial ruin by an outbreak of surra among the animals used for transport in that country. Trypanosome diseases have also been the subject of an address by Koch[16] in Berlin. Classed among the flagellata or ciliated protozoa trypanosomes resemble in shape a fish two or three times the length of a red blood corpuscle, having a long flagella, an undulating membrane on one side, a large nucleus, and a smaller nucleus or centroscme. They increase by longitudinal fission, show very active movements and swim with the flagella end first. The features of disease common to trypanosome infection in many respects resemble those produced by the malarial parasite. The illness, occasionally acute but far more often chronic, is characterised by prolonged, irregular, and intermittent fever, wasting, anæmia, weakness, localised œdema, and enlargement of the spleen and lymphatic glands. Authorities differ as to the classification of the trypanosomes known at present. Laveran and Mesnil[17] lay stress on the contention that those found in the different diseases are specifically distinct. Koch, on the other hand, would divide them into two groups: one containing the trypanosome of Lewis found in rats and that of Theiler found in cattle; the other containing those of tsetse-fly disease, surra, mal de caderas, and the trypanosomiases of man—namely, trypanosomiasis and sleeping sickness, seen in Africa, the spotted fever of North America, tropical splenomegaly and Oriental (Delhi, Aleppo, or Biskra) boil. Those of the second group are morphologically indistinguishable in Koch's opinion, but vary greatly in virulence and in size and form according to the animal into which they have been inoculated. The attempts to secure immunity by inoculation with attenuated strains of virulent infection have proved disappointing. Thongh the animals inoculated may enjoy an apparent immunity they may still harbour the parasites in their blood and thus become a source of infection to others. Such artificially immunised animals would be in the same position as the large antelopes and buffaloes which, according to those most experienced in the tsetse regions, are the principal source of tsetse infection. Hence artificial immunisation of cattle is deemed inadvisable, but since tsetse flies and tsetse disease disappear with the destruction of big game, cattle may perhaps be saved by clearing off such game. Both cannot live together on African soil. Isolation might be employed in the case of sufferers from sleeping sickness, but this again would be useless because numbers of apparently healthy natives harbour the parasite in their blood. In the future a drug may be found as efficacious as is quinine in malaria. Arsenic appears to be of some slight benefit, and a remedy discovered by Ehrlich, called trypanroth, has given better results, especially if combined with arsenic. C. Christy[18] thinks the most important prophylactic step is to thoroughly investigate and map out the "fly" area. He thinks that probably the area of distribution of *Glossina palpalis* does not extend more than a mile from the shores of Lake Victoria Nyanza. He lays stress on the fact that measures applicable, *e.g.*, to the staying of plague are inapplicable to sleeping sickness on account of the disease being frequently latent and only to be diagnosed by repeated, lengthy, and skilled observations. He draws attention to the appearance of the blood-film in infected cases. Clumping or agglutination of the red cells is visible even to the naked eye. In the Soudan *Glossina palpalis* has not been found, but A. Balfour and Bimbashi Head[19] have found trypanosomiasis in cattle in the Southern regions. In India, where the tsetse fly is unknown, Rogers has proved that the horsefly can convey the trypanosome of surra.[20]

[8] Brit. Med. Jour., p. 646. [9] Ibid., p. 653. [10] Lancet, Sept. 10. [11] Brit. Med. Jour., p. 644. [12] Prac., Nov. [13] Brit. Med. Jour. Sept. 17, p. 648. [14] Prac., Nov., p. 676. [15] Brit. Med. Jour., Oct. 15. [16] Ibid., Nov. 26. [17] Ibid., p. 1478. [18] Ibid., p. 1457. [19] Ibid., p. 1455. [20] Ibid.

(*To be continued.*)

